# Lewis Pair Polymerization for New Reactivity and Structure in Polymer Synthesis

**DOI:** 10.3390/molecules23040915

**Published:** 2018-04-16

**Authors:** Jiawei Chen, Miao Hong, Eugene Y.-X. Chen

**Affiliations:** 1 Department of Chemistry, Columbia University, 3000 Broadway, New York, NY 10027, USA; 2 State Key Laboratory of Organometallic Chemistry, Shanghai Institute of Organic Chemistry, Chinese Academy of Sciences, Shanghai 200032, China; 3 Department of Chemistry, Colorado State University, Fort Collins, CO 80523, USA

The reactivity of a Lewis pair has been mainly judged by its ability to form a Lewis adduct or not, according to the Lewis definition [1]. However, the discovery of reversible dihydrogen activation in 2006 enabled by the so-called frustrated Lewis pair (FLP) that is sterically separated but still possesses highly unquenched, orthogonal Lewis acid and base reactivity has changed the view of the reaction pattern of a Lewis pair [2]. Thanks to intensive research carried out on this nonclassical Lewis pair reactivity, FLP chemistry has been developed into a powerful tool in small molecule activation, organic transformation, and polymer synthesis over the years. In 2010, Lewis pair polymerization (LPP) [3], which employs an FLP, a classical Lewis adduct (CLA), or an interacting Lewis pair, emerged to promote cooperative monomer activation and chain initiation and/or propagation, the cooperativity of which was exploited for the polymerization of polar and non-polar vinyl monomers as well as heterocyclic monomers to afford a variety of polymer structures [4]. Herein, with the aim of updating and highlighting some recent developments in the area of LPP, we have gathered 10 articles in this Special Issue entitled “Lewis Pair Polymerization for New Reactivity and Structure in Polymer Synthesis” under the following three major topics.

Firstly, the studies on the controlled or chemoselective addition polymerizations of conjugated polar alkene monomers represent four articles in this Special Issue [5,6,7,8]. Specifically, the controlled and efficient polymerizations of polar monomers such as methyl methacrylate (MMA), *n*-butyl methacrylate (*n*BMA), and *γ*-methyl-*α*-methylene-*γ*-butyrolactone (*γ*MMBL) catalyzed by the sterically hindered aryloxide-substituted alkylaluminum/*N*-heterocyclic carbene (NHC) Lewis acid/base catalyst system were reported by Hong and co-workers [5]. Zhang and He employed the silyl ketene acetal/B(C_6_F_5_)_3_ Lewis pair system to render the living group-transfer polymerization of a similar monomer scope, including linear MMA and cyclic renewable acrylics (*α*-methylene-*γ*-butyrolactone and *γ*MMBL) [6]. Xu’s group further reported the chemoselective polymerization of polar divinyl monomers such as allyl methacrylate, vinyl methacrylate, and 4-vinylbenzyl methacrylate using simple homoleptic rare-earth/phosphine Lewis pairs, as well as the post modifications of the retained side-chain double bonds with the thio-ene reaction [7]. The ability to bring about chemoselective polymerization by Lewis pairs was also exploited Lu and co-workers to synthesize PMMA-based copolymers with complex structures [8]. Thus, the installation of pendant vinyl groups onto the NHC catalyst or monomer led to two types of polymers bearing –OH groups through the thio-ene click functionalization; these macro-initiators were then utilized to promote the ring-opening polymerization of lactide to produce block and brush copolymers [8].

Secondly, another major topic of the contributions collected in this issue is ring-opening polymerization. The current progresses on the Lewis pair-mediated ring-opening polymerization of lactide and related cyclic esters were reviewed by Wu and co-workers [9]. Naumann’s group examined the mechanism of lactone polymerization enabled by *N*-Heterocyclic olefins paired with different halides as Lewis acids, including ZnCl_2_, MgCl_2_, and LiCl, through a computational study [10]. In addition, Yang and Du reported the synthesis of well-defined polypeptides from the ring-opening polymerization of *N*-carboxyanhydrides catalyzed by zinc acetate Zn(OAc)_2_ using a variety of anilines as the base [11]. The copolymerization of carbonyl sulfide and propylene oxide in the presence of polyethylene glycol to yield block copolymers with perfectly alternating and regio-regular poly(monothiocarbonate) segments was described by Zhang [12]. Narrow molecular weight distributions and high turn-over frequencies were achieved by the applied metal-free Lewis pair catalyst systems in conjunction with different chain transfer agents [12].

Thirdly, constructions of complex and interesting polymeric or supramolecular structures, through the modulation of Lewis acid-base interactions on the macromolecular level, were accomplished by the groups of Chen and Jäkle. Chen’s team demonstrated that poly(3-hexylthiophene) macromer anchored with alkyl or vinyl imidazolium end groups can be readily converted into a polymeric NHC Lewis base that is capable of binding to Lewis acidic C_60_ to form a single donor-acceptor dyad or brush of donor-acceptor dyads. The special architecture of brush donor-acceptor dyads provides promising potential applications in polymer-based solar cells [13]. In a different way, Jäkle and co-workers reported the interaction pattern between a polymeric Lewis acid and a telechelic Lewis base [14]. The Lewis acidic polymer was obtained by the partial decoration of the para-phenyl position of polystyrene with electron-deficient borane moieties, whereas the Lewis basic polymer was synthesized by capping polydimethylsiloxane with electron-donating pyridine units at both ends. The mixing of such Lewis acidic and basic polymers results in the formation of solid-like gel with a crosslinked network that exhibits dynamic properties and thermally induced self-healing behaviors [14].

Finally, the guest editors of this Special Issue wish to take this opportunity to thank all of the authors who contributed to this Special Issue. We also hope the readers will enjoy reading this first thematic issue on LPP, which covers a broad spectrum of topics on the exploration of Lewis pair catalyst/initiator and monomer scopes, the synthesis of polymers with controlled or unique structures through catalyst design and Lewis interaction modulation, as well as the related mechanistic investigations through both experimental and computational studies.
